# Maternal effects on the development of vocal communication in wild chimpanzees

**DOI:** 10.1016/j.isci.2022.105152

**Published:** 2022-09-19

**Authors:** Aisha C. Bründl, Cédric Girard-Buttoz, Tatiana Bortolato, Liran Samuni, Mathilde Grampp, Therese Löhrich, Patrick Tkaczynski, Roman M. Wittig, Catherine Crockford

**Affiliations:** 1 Department of Human Behavior, Ecology and Culture, Max Planck Institute for Evolutionary Anthropology, Leipzig 04103, Germany; 2 Department of Neuropsychology, Max Planck Institute for Human Cognitive and Brain Sciences, Leipzig 04103, Germany; 3 Taï Chimpanzee Project, Centre Suisse de Recherches Scientifiques, 01 BP 1303, Abidjan, Côte d’Ivoire; 4 The Great Ape Social Mind Lab, Institut des Sciences Cognitives, CNRS, 67 Boulevard Pinel, Bron, 69675 Lyon, France; 5 Department of Human Evolutionary Biology, Harvard University, Cambridge, MA 02138, USA; 6 Epidemiology of Highly Pathogenic Microorganisms, Robert Koch Institute, Seestraße 10, Berlin 13353, Germany; 7 World Wide Fund for Nature, Dzanga Sangha Protected Areas, Bangui BP 1053, Central African Republic; 8 Veterinary Group Practice Heeslingen, Stader Straße 5, 27404 Heeslingen, Germany; 9 School of Biological and Environmental Sciences, Liverpool John Moores University, Liverpool L33AF, UK

**Keywords:** Biological sciences, Zoology, Ethology

## Abstract

Early-life experiences, such as maternal care received, influence adult social integration and survival. We examine what changes to social behavior through ontogeny lead to these lifelong effects, particularly whether early-life maternal environment impacts the development of social communication. Chimpanzees experience prolonged social communication development. Focusing on a central communicative trait, the “pant-hoot” contact call used to solicit social engagement, we collected cross-sectional data on wild chimpanzees (52 immatures and 36 mothers). We assessed early-life socioecological impacts on pant-hoot rates across development, specifically: mothers’ gregariousness, age, pant-hoot rates and dominance rank, maternal loss, and food availability, controlling for current maternal effects. We found that early-life maternal gregariousness correlated positively with offspring pant-hoot rates, while maternal loss led to reduced pant-hoot rates across development. Males had steeper developmental trajectories in pant-hoot rates than females. We demonstrate the impact of maternal effects on developmental trajectories of a rarely investigated social trait, vocal production.

## Highlights

• Wild chimpanzees show individual variation in pant-hoot rate development
• Males have steeper developmental trajectories in pant-hoot rates than females
• Immature pant-hoot rates correlate with early-life maternal gregariousness
• Maternal loss leads to reduced pant-hoot rates across development

## Graphical abstract

**Figure:**
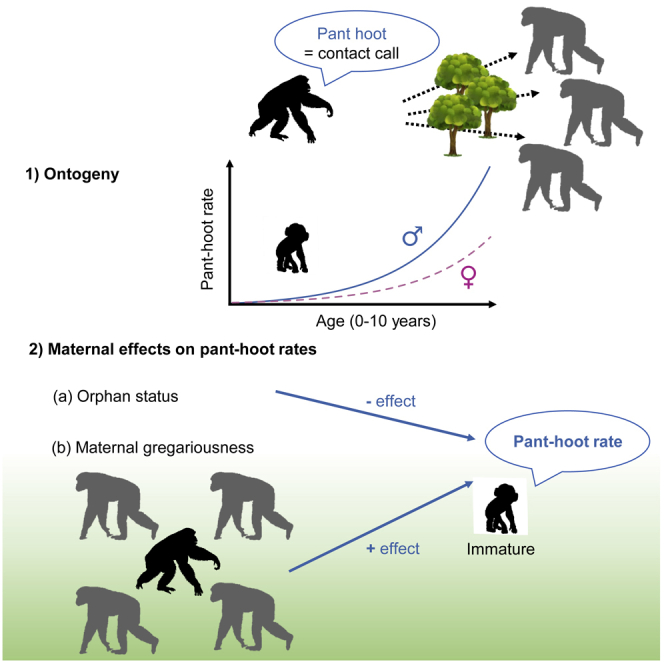

## Introduction

Maternal effects, including maternal care, can be crucial in shaping sociality in mammalian species that depend heavily on post-natal maternal care (Clutton-Brock, 1991; Crockford et al., 2020). At the extreme, maternal loss can have long-lasting, negative impacts on social integration and fitness correlates, such as survival, for current and following generations (e.g., in elephants (Goldenberg and Wittemyer, 2017), baboons (Tung et al., 2016; Zipple et al., 2019), chimpanzees (Kalcher-Sommersguter et al., 2015; Samuni et al., 2020a), primates (Zipple et al., 2021), and social mammals including humans (Snyder-Mackler et al., 2020)). Nevertheless, little is known about the underlying mechanisms involved, such as how early maternal effects modify offspring social behavior resulting in negative outcomes such as social alienation in adult life. Studies assessing the impact of maternal effects on social behaviors critical for building and managing relationships have principally examined grooming, agonism, and association patterns in adults (e.g., in hyaenas (Dloniak et al., 2006; Holekamp et al., 1997), mice (Kikusui et al., 2005), and bonobos (Surbeck et al., 2011), reviewed in: Clutton-Brock, 2016). Fewer studies have examined potential maternal effects on social behavior during ontogeny (e.g., in chimpanzees (Markham et al., 2015; van Leeuwen et al., 2014) and humans (Christoffersen, 2012)) and, in particular, on vocal communication.

Communication is a critical dimension of sociality in several animal species since, across signaling modalities, it is often the negotiation pathway that facilitates social contact such as grooming or agonism (Laidre and Johnstone, 2013). Hence, examining the impact of maternal effects on signal production during ontogeny might give insight into *how* mothers impact the sociality of their offspring. In particular, the impact of mothers and fathers on vocal learning of song is well studied (in birds and mammals (Janik and Slater, 2000; Snowdon and Hausberger, 1997)). Far fewer studies have examined the parental impact on the development of vocal signals that navigate social interactions (for birds and mammals see: Hollén et al., 2008; Hollén and Radford, 2009; Janik and Slater, 1997; Seyfarth and Cheney, 1980, 2010). Rates of other social behaviors, for example, grooming and play, are used as key indicators of early-life maternal effects (e.g., in rodents (Auger and Olesen, 2009; Kikusui et al., 2005)), but rates in terms of communication are overlooked. In our study, we address whether early-life maternal effects impact production rates of a communicative behavior key in contact maintenance.

Maternal effects on social behavior, in general, can be examined by measuring the relationship between maternal characteristics and offspring social behavior. Maternal traits such as grooming rates toward their offspring and general gregariousness positively impact social traits of the offspring during development and upon reaching adulthood (e.g., in rodents (Francis et al., 1999), dolphins (Gibson and Mann, 2008), and orang-utans (Fröhlich et al., 2020)). Alternatively, maternal effects can be quantified by observing the impact of developmental disruptions, such as maternal loss, on social behavior (e.g., in rodents and primates (Grampp et al., 2019; Sánchez et al., 2001)). For example, in captivity, chimpanzees orphaned early in life show lower grooming activity than non-orphaned chimpanzees, resulting in decreased social integration as adults (Kalcher-Sommersguter et al., 2015). Similarly, maternal absence leads to immature chimpanzees engaging in shorter play bouts that more frequently end in aggression as compared to their counterparts with mothers (van Leeuwen et al., 2014). Human children that experience early-life separation from their natal families and grow up in institutions demonstrate significantly poorer cognitive and social development compared to children raised in a family environment (Christoffersen, 2012). Thus, we hypothesize that in species reliant on parental care, parents’ sociability during early life will have a potentially large impact not only on the offspring’s social interactions but also on the offspring’s motivation to socialize and therefore to communicate with conspecifics (Cheney and Seyfarth, 2018).

Communication rates, such as vocal production rates, may be a means to quantify the impact of early-life experience on later social motivation but this remains poorly investigated. Here, we examine maternal effects on vocal output and more specifically on the production of a contact call. Contact calls are particularly relevant vocal traits since they are widespread in the animal kingdom (reviewed in: Sewall et al., 2016). They are often individually distinctive and are used by many social animals to indicate their location to group mates and seek contact with others over variable distances (e.g., in meerkats (Engesser and Manser, 2022), non-human primates (Cheney and Seyfarth, 1996), and see review of bird and mammal studies: Kondo and Watanabe, 2009). Hence, they might operate as an indicator of social motivation across many species (e.g., in bats (Arnold and Wilkinson, 2011) and macaques (Suzuki and Sugiura, 2010), see review in primate species: Cheney and Seyfarth, 2018). For example, in baboons, grunt contact calls from dominants to subordinates act as a signal of benign intent during approaches, increasing the likelihood that subordinates tolerate dominant approaches (Silk et al., 2016). In African elephants, long-distance contact calls have also been shown to mediate social interactions resulting in approaches between affiliated dyads (Leighty et al., 2008). Few studies have investigated early-life maternal effects on individual variation in vocal development of social calls in non-singing species. Examples include vocal convergence in goats; half-siblings showed more similar calls when raised in the same compared to different social groups (Briefer and McElligott, 2012). Parental interaction effects on the development of vocal structure in marmosets have been demonstrated; i.e., those with limited parental interactions expressed less mature patterns in acoustic structure and duration of the long-distance contact “phee” call compared to normally raised individuals (Gultekin and Hage, 2018). Also, studies on vocal sequence flexibility in sub-adult gibbons with observed acoustic matching between mothers and daughters (Koda et al., 2013), and alarm call development in birds and mammals (reviewed in: Hollén and Radford, 2009) highlight such early-life effects on vocal development. Vocal development can also be affected by various non-maternal early-life effects, such as social exposure to conspecifics: group size influences the development of sentinel call rates in wild meerkats, such that immature individuals raised in smaller groups begin calling sooner during ontogeny than those from larger groups (Rauber and Manser, 2021). However, for many mammal species, mothers are the main social partner for much of development, and thus also the main determinant of the immediate social environment of their offspring (Broad et al., 2006; Mateo, 2009).

In this study, we examined individual variation in the development of the rate of a primary contact call, the chimpanzee pant hoot. Chimpanzees have a prolonged development where maternal effects on social (Kalcher-Sommersguter et al., 2015; Markham et al., 2015; van Leeuwen et al., 2014), but not vocal behavior have been identified. The pant hoot is a long-distance contact call that carries distances of at least 500 m (Ghiglieri, 1984; Kalan et al., 2016) and encodes information about the caller identity (Crockford et al., 2004; Desai et al., 2021; Marler and Hobbett, 1975; Mitani et al., 1996). Pant hoots likely function to maintain contact between subgroups of community members, particularly with allies (Goodall, 1986; Marler and Hobbett, 1975), and may function to recruit allies (Kalan and Boesch, 2015) in this fission-fusion species, in which group composition and size change frequently over time (Ramos-Fernández and Morales, 2014). To our knowledge, studies investigating early-life sources of individual variation in contact call rates across development in long-lived mammals are rare but may provide an additional useful social metric for assessing the impact of maternal effects on social development.

We tested early-life maternal effects that are expected to influence the trajectory of pant-hoot rates during development. An advantage of examining the pant hoot is that it is a complex call often including a vocal sequence of four or more call types; “introductory” hoos, “build-up” panted hoos, “climax” phase, hoos or barks, and “let-down” panted roars (Arcadi, 1996; Crockford and Boesch, 2005; Girard-Buttoz et al., 2022b). Furthermore, pant hoos are central calls in chimpanzee communication since they are frequently combined with other call types such as the submissive pant grunt (Girard-Buttoz et al., 2022b) or food calls (Leroux et al., 2021) in several vocal sequences (Girard-Buttoz et al., 2022b). As this complex call likely requires maturational processes at neurological, respiratory, or articulatory levels to be produced, we do not expect this call to be expressed at the start of life. This is supported by previous findings demonstrating that behavioral traits including communication develop slowly across ontogeny in chimpanzees (Bründl et al., 2021) with this call emerging before puberty (Bortolato et al., 2022 in revision). Males pant hoot more frequently as adults than females (Crunchant et al., 2021; Kalan, 2019), though this call is emitted by both sexes to maintain contact with and recruit group members (Clark, 1993). Overall, this communication trait thus lends itself well to investigating early-life maternal effects on social development.

Chimpanzees are dependent on maternal care at least until weaning at 3–6 years of age (Lonsdorf et al., 2020) but typically continue to associate with mothers at high rates until adolescence, after 10 years of age (Pusey, 1983; Reddy and Sandel, 2020). Thus, maternal gregariousness, dominance rank, age, and social behaviors likely influence the social exposure of offspring for at least the first decade of life, potentially impacting offspring social motivation to engage with others. The first years of life are particularly influential in chimpanzees as offspring are still fully dependent on their mothers (Lonsdorf et al., 2020) and crucial developmental milestones including social traits are reached during this period (Bründl et al., 2021). Consequently, our first prediction (P1) is that offspring born to a mother that is highly gregarious (e.g., more reason to produce pant hoots in order to coordinate party movements), high-ranking (linked to higher pant-hoot rates (Clark, 1993; Mitani and Nishida, 1993)), older (a proxy for maternal experience), and/or that produced pant hoots frequently during their first two years of life will have a higher rate of pant hooting than other offspring. In the second part of our study, we focused on a more extreme maternal effect, maternal loss. Maternal loss is costly for offspring chimpanzees since offspring who lose their mothers before adulthood have reduced growth (Samuni et al., 2020b), survival (Nakamura et al., 2014; Stanton et al., 2020), and reproductive success (Crockford et al., 2020) compared to those with mothers. In our study, we measured one type of potential social cost of maternal loss, namely reduced communication, which is a key mediator of social interactions. Specifically, we tested whether maternal loss impacts vocal production rates by investigating the calling pattern of orphans in comparison to non-orphans. Our second prediction was that orphans will call less often than non-orphans (P2), due to a possible lack of social engagement, integration, and motivation associated with having no mother to call to/with and/or experiencing fear of calling. This fear may be due to loud pant hoots advertising an individual’s location (Fedurek et al., 2014), which can theoretically lead to increased rates of received aggression. Orphans may be especially vulnerable to receiving aggressions without the protection of a mother (Reddy and Mitani, 2019).

It has also been shown that early-life ecological factors affect developmental trajectories in primates (Lummaa and Clutton-Brock, 2002; Tung et al., 2016; Zipple et al., 2019). Thus, we tested an alternative ecological hypothesis to the maternal hypotheses. Chimpanzees rely heavily on ephemeral ripe fruits for nutrients, with availability differing among seasons and forest type (primary versus secondary rainforest (Gone Bi and Wittig, 2019; Wessling et al., 2018)). Sufficient fruit availability in a territory may enhance chimpanzee immatures’ condition, physical maturation, and enhance investment in the development of social traits (Tkaczynski et al., 2020b). Thus, our third prediction (P3) is that immature chimpanzees growing up with high compared with low fruit availability during their first two years of life can invest more in social activity and will pant hoot at higher rates.

## Results

### Early-life factors

**Figure 1:**
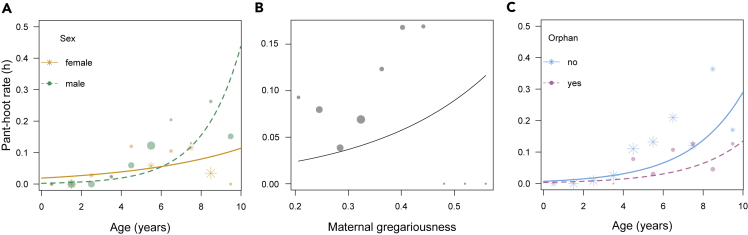
Early-life effects on immature chimpanzees’ pant-hoot rate The effect on immature chimpanzees’ pant-hoot rate (h), aged 0–10 years (N = 792), of (A) sex; (B) maternal gregariousness and (C) orphan status. The predictor values are binned for (A) and (C) per year and for (B) by 0.05 increments of maternal gregariousness. The points show raw values with the size representing the sample size, while the predicted lines are based on the fitted model values from the “early-life model” (Table 2).

We observed immature chimpanzees (i.e., aged 0–10 years) first pant hooting at 2.6 years and an increase in pant-hoot rate with age (Figure 1A). Our “early-life” full model testing what early-life predictors influence pant-hoot rate, while controlling for current FAI (food availability index), current number of siblings, and current number of adults in the party, (the exact definition of each variable included in the models can be found in Table 1) was significantly different from the null model (LRT: *χ*^*2*^ = 30.764, *DF* = 12; p = 0.002), suggesting independent or interacting effects of immature age and sex, orphan status, early-life maternal factors, and variation in food availability (food availability index (FAI)). After removing the non-significant two-way interactions between age and early-life maternal predictors, age and orphan status (LRT, all p > 0.100), we found a significant interaction between age and sex (LRT, p = 0.002; Table 2). Males showed a three times steeper increase in pant-hoot rates with age than females (Figure 1A). Early-life maternal gregariousness had a significant positive effect on immature pant-hoot rate production (LRT, p = 0.014; Figure 1B and Table 2). There was also a significant negative effect of maternal loss, with orphans uttering on average 20% fewer pant hoots than non-orphans throughout ontogeny (i.e., after controlling for age, p = 0.006; Figure 1C and Table 2). The average pant-hoot rates were mean ± SD 0.08 ± 0.19 vs. 0.10 ± 0.27 pant hoots per hour for orphans and non-orphans, respectively. The following fixed effects: early-life maternal factors of age, dominance rank, pant-hoot rate, and early-life FAI, were all non-significant (Table 2). Out of all the control predictors, only “*current party size*” showed a positive effect on immatures pant hooting (LRT, p = 0.047; Table 2). In this model, the proportion of variance in the response explained by the fixed effects only was R^2^_m_ = 0.25 and by the random and fixed effects was R^2^_c_ = 0.39.

**Table 1:** Definition of the variables included in the models

Predictors	Definition
**Test predictors**	

Focal age	Age (in days) on the focal observation day
Sex	Female or male
Orphan status	If orphaned or not (after a minimum of 1 month after maternal loss)
Early-life maternal gregariousness	Average number of adults in the maternal party divided by the total number of adults in the community across the early-life window
Early-life maternal age	Maternal age (in days) on the birth date of the focal
Early-life maternal dominance rank	Average maternal dominance rank across the early-life window
Early-life maternal pant-hoot rate	Maternal sum of pant hoots divided by the maternal sum of observation time (in hours) across the early-life window
Early-life FAI	Average food availability index (FAI) across the early-life window
Current maternal presence	If the mother was present or not in the focal’s party
Current maternal gregariousness	Average number of adults in the maternal party divided by the total number of adults in the community across the year of the focal observation day
Current maternal dominance rank	Maternal dominance rank on the focal observation day
Current maternal pant-hoot rate	Maternal sum of pant hoots divided by the maternal sum of observation time (in hours) averaged across the year of the focal observation day

**Control predictors**	

Current FAI	FAI in the month of the focal observation day
Current party size	Number of adults in the focal’s party divided by the total number of adults in the community
Current number of older siblings	Number of older siblings of the focal

The early-life window encompasses the time period for each individual between birth and the end of the first two years of life, except for individuals that were younger than two years—here the endpoint was the last observation date. The currnt window is detailed for each variable.

**Table 2:** Early-life effects on pant-hoot rates from the reduced GLMM with negative binomial error structure and logit link function (N = 792)

Predictors	Reference category	Estimate	± SE	Lower 95% CI	Upper 95% CI	*Z* value	*X*^*2*^	p value
Intercept		**−2.907**	**0.334**	**−3.620**	**−2.361**	**−8.702**	–	–

**Test predictors**								

Focal age		0.421	0.231	0.002	0.829	1.821	–	–
Sex (Male)	Female	−0.378	0.225	−0.825	0.082	−1.681	–	–
Orphan status (Yes)	No	**−0.765**	**0.277**	**−1.410**	**−0.186**	**−2.766**	**7.696**	**0.006**
Early-life maternal gregariousness		**0.300**	**0.123**	**0.028**	**0.566**	**2.448**	**6.016**	**0.014**
Early-life maternal age		−0.020	0.111	−0.313	0.227	−0.184	0.071	0.790
Early-life maternal dominance rank		0.076	0.151	−0.212	0.403	0.505	0.258	0.612
Early-life maternal pant-hoot rate		−0.275	0.198	−0.753	0.051	−1.387	2.085	0.149
Early-life FAI		0.036	0.170	−0.345	0.359	0.213	0.046	0.830
Focal age∗Sex (Male)	Female	**0.804**	**0.251**	**0.338**	**1.239**	**3.208**	**9.831**	**0.001**

**Control predictors**								

Current FAI		0.046	0.136	−0.206	0.263	0.341	0.108	0.743
Current party size		**0.227**	**0.116**	**−0.020**	**0.440**	**1.956**	**3.961**	**0.047**
Current number of older siblings		−0.051	0.150	−0.362	0.239	−0.340	0.146	0.703

Statistically significant effects (p ≤ 0.05) appear in bold and the coded level of factors in parenthesis. p values are derived from likelihood ratio tests based on chi-square (X2) values. X2 and p values are not indicated in the first three rows because of having a very limited interpretation. All continuous predictors are z-transformed to a mean of 0 and a standard deviation of 1. Degrees of freedom are 1 for all predictors.

### Current maternal factors

In the “current maternal model”, the full model did not significantly differ from the null model (likelihood-ratio test: *χ*^*2*^ = 2.210, *DF* = 4; p = 0.697), suggesting that no current maternal factors (i.e., maternal gregariousness, pant-hoot rates, dominance rank, and presence) significantly affected the immature chimpanzees’ pant-hoot rates (Table 3), even though the mother was present in 98% of the current immatures’ parties (see Figure S3). These non-significant results held true after we ran a post-hoc model removing all early-life variables (see Table S2).

**Table 3:** Current maternal effects on pant-hoot rates from the reduced GLMM with negative binomial error structure and logit link function (N = 595)

Predictors	Reference category	Estimate	± SE	Lower 95% CI	Upper 95% CI	*Z* value	*X*^*2*^	p value
Intercept		**−2.944**	**0.289**	**−3.700**	**−2.509**	**−10.183**	–	–

**Test predictors**								

Focal age		**1.068**	**0.226**	**0.662**	**1.548**	**4.735**	**22.063**	**<0.001**
Sex (Male)	Female	−0.513	0.311	−1.119	0.136	−1.653	2.822	0.093
Early-life maternal gregariousness		**0.322**	**0.150**	**−0.037**	**0.641**	**2.151**	**4.250**	**0.039**
Early-life maternal age		−0.034	0.262	−0.573	0.432	−0.130	0.039	0.844
Early-life maternal dominance rank		0.065	0.190	−0.299	0.434	0.344	0.142	0.706
Early-life maternal pant-hoot rate		−0.209	0.264	−0.851	0.195	−0.792	0.667	0.414
Early-life FAI		0.005	0.182	−0.441	0.337	0.030	0.017	0.898
Current maternal presence		0.032	0.104	−0.095	0.626	0.309	0.099	0.753
Current maternal gregariousness		−0.125	0.149	−0.453	0.146	−0.838	0.732	0.392
Current maternal dominance rank		0.171	0.272	−0.347	0.748	0.629	0.397	0.529
Current maternal pant-hoot rate		0.158	0.100	−0.127	0.366	1.587	2.211	0.137

**Control predictors**								

Current FAI		−0.011	0.181	−0.367	0.267	−0.059	0.005	0.944
Current party size		**0.261**	**0.134**	**−0.013**	**0.509**	**1.954**	**3.848**	**0.050**
Current number of older siblings		0.002	0.191	−0.392	0.427	0.013	0.019	0.891

Statistically significant effects (p ≤ 0.05) appear in bold and the coded level of factors in parenthesis. p values are derived from likelihood ratio tests based on chi-square (*X*^*2*^) values. *X*^*2*^ and p values are not indicated in the first row because of having a very limited interpretation. All continuous predictors are z-transformed to a mean of 0 and a standard deviation of 1. Degrees of freedom are 1 for all predictors.

## Discussion

This study investigated the effects of early-life maternal effects on rates of production of a highly social contact call—the pant hoot—in immature wild chimpanzees. We first observed pant hoots produced after 2.6 years of age with a gradual increase in production rate with age (Figure 1A). We identified maternal effects on social call production. Offspring with highly gregarious mothers during their early ontogeny had higher pant-hoot rates than offspring whose mothers were less social in the first two years of life. Our model suggests that these effects were independent of the mothers’ pant-hoot rates in the same period when offspring were sampled as well as current maternal pant-hoot rates and gregariousness. Maternal effects were also revealed in our orphan-non-orphan comparison, with orphans calling less frequently than non-orphans. Finally, immature chimpanzees pant hooted more frequently when in the presence of more adults, indicating an effect of the current social dynamic.

Maternal gregariousness during early life had a positive effect on offspring pant-hoot rates, independent of current party size and current maternal pant-hoot rates. Maternal gregariousness in early life has been shown to shape social development in other species with both fast and slow life histories (e.g., in rodents (Francis et al., 1999), dolphins (Gibson and Mann, 2008), and orang-utans (Fröhlich et al., 2020)). Chimpanzee offspring have a prolonged juvenile dependency on mothers, which includes mothers being their primary social partner prior to the onset of sexual maturity (Reddy and Sandel, 2020). As such, maternal gregariousness has a primary influence on social exposure in this species. Such early-life social exposure may be directly linked to opportunities for social learning and skill acquisition (Schuppli et al., 2020). While adult female gregariousness is a relatively stable trait in the Taï chimpanzee population (Tkaczynski et al., 2020b), it nonetheless likely varies over offspring ontogeny according to resource availability, presence of estrous females (Anderson et al., 2002; Wittiger and Boesch, 2013), reproductive state of the mother (Goodall, 1986; Matsumoto-Oda, 1999), and the developmental phase of the offspring (e.g., Murray et al., 2014; Otali and Gilchrist, 2006). For example, Eastern chimpanzee mothers are less gregarious than non-mothers, especially in the presence of males (Otali and Gilchrist, 2006). Furthermore, Eastern chimpanzee mothers with infant sons are more gregarious than those that have infant daughters, in particular during the first 6 months after birth (Murray et al., 2014), highlighting some temporal flexibility in mothers’ gregariousness. The absence of an effect of current maternal gregariousness on immature calling rates may thus be due to the “current maternal gregariousness” variable being binned within a year (to reduce measurement error by maximizing data availability as offspring and mothers were observed during different time periods) even though party size changes a lot within a year resulting in independent variation in immature pant-hoot rates. Also, after two years of age, current maternal gregariousness becomes less important as offspring take more agency in their vocalizing based on current social circumstances. This may indicate that offspring start developing their own social phenotype independent of their mother’s social phenotype before reaching independence. Further studies should investigate other social effects that may be linked to maternal gregariousness such as maternal social integration, bondedness, or dyadic association patterns, which may all affect social exposure of offspring (Murray et al., 2014). In addition, social dynamics of the group such as party composition (Soldati et al., 2022), fission-fusion, and pant-hoot rates by other group members may play a role in shaping vocal development in immature chimpanzees.

When examining the impact of maternal loss, we found that orphans called at a lower rate compared to non-orphans. The effect of age on pant-hoot rates did not differ significantly between orphans and non-orphans. It is important to consider that our data included orphans from four years old onward as chimpanzees rarely survive without a mother before this age. Thus, these chimpanzees were orphaned after the two-year early-life window, indicating that current factors (rather than early-life factors) such as physiology, energetics, and competition may drive the call production differences observed between orphans and non-orphans. A recent study revealed that weaned chimpanzee males, orphaned before reaching maturity, started reproduction later and experienced decreased reproductive success as compared to non-orphaned individuals (Crockford et al., 2020). Furthermore, during development, orphaned chimpanzees exhibit lower muscle mass than non-orphans (Samuni et al., 2020b) and have cortisol levels indicative of exposure to nutritional stress (Girard-Buttoz et al., 2021). Lower mass and exposure to nutritional stress may be associated with delayed development of other traits (Hediger et al., 2002; Stanton et al., 2020). Whether delayed social development potentially contributes to the lower rates of pant hoots observed in orphans is a question for future studies.

A lower contact call rate through development may also indicate a lower social integration of orphans versus non-orphans. This would be important to assess in future studies, as maternal loss is known to negatively impact social integration in several social mammals including hyenas, elephants, baboons, chimpanzees, and humans (Goldenberg and Wittemyer, 2017; Kalcher-Sommersguter et al., 2015; Snyder-Mackler et al., 2020; Strauss et al., 2020; Tung et al., 2016). Lower social integration and bonding capacity may be in turn linked to decreased social motivation. For instance, in chimpanzees, maternal loss may remove social buffering against aggressions received (Miller et al., 2017). Associating with others may thus carry higher risks for orphans and result in lower social motivation to associate, and hence lower likelihood to produce contact calls. Pant hoots are often chorused, meaning that chimpanzees simultaneously pant hoot (Arcadi, 1996). Chimpanzees have been shown to pant hoot more frequently together than individually, in particular with preferred long-term social partners (Fedurek et al., 2013). Orphan chimpanzees may thus miss out on having a key chorusing partner, i.e., their mothers, leading to lower call frequencies. In our study, current maternal pant-hoot rates did not influence pant-hoot rates of the immatures, though here we did not examine chorusing *per se*. To help disentangle the various mechanisms that led to the observed lower rates of calling in orphans, more data in a greater sample of orphans and non-orphans in different social contexts are needed.

Pant-hoot production emerged in both sexes well in advance of adolescence suggesting that pant-hoot rates are not solely a sexually selected trait. However, immature males had a steeper developmental increase in pant-hoot rates than females consistent with the possibility that sexual selection nevertheless plays a role in shaping pant-hoot production in chimpanzees (Figure 1A). The sex-specific trajectories of pant hoot call rates in our study mirror similar sex differences in ontogeny observed in the chimpanzee literature: males not only grow faster (Samuni et al., 2020b) but also socialize outside the mother-offspring dyads earlier in ontogeny (Lonsdorf et al., 2014). In the Taï population, adult male chimpanzees pant hoot almost three times as much as adult females (1.23 vs. 0.46 pant hoots per hour on average (Kalan, 2019)). In other populations, higher-ranking males also pant hoot more frequently than subordinate males (Clark, 1993; Mitani and Nishida, 1993). Therefore, our results regarding sex differences are broadly in keeping with expectations for a species with male philopatry, where males may gain greater reproductive benefits from maintaining community cohesion and calling more frequently (Lemoine et al., 2020; Pusey and Parker, 1987; Samuni et al., 2020b).

We found no significant effect of early-life maternal factors of age, dominance rank, pant-hoot rate, and early-life food availability on the pant-hoot rate of our study individuals. In primary rainforests, where food is generally plentiful throughout the year, maternal effects such as gregariousness may have a much stronger effect than early-life ecological impacts such as variation in FAI.

## Conclusions

In conclusion, pant-hoot calling rates in immature wild chimpanzees were predicted by both individual factors (age and sex) and maternal effects (gregariousness and overall presence of the mother) in early life. Additionally, immature chimpanzees pant hooted more frequently when in the presence of more adults, indicating an influence of current social dynamics. Our study highlights the long reach of the early maternal environment on chimpanzee vocal production rates later in life. These results suggest first, that vocalizations, such as contact calls, can be an additional social measure, along with grooming, association, and aggression rates, to quantify social motivation to engage with conspecifics throughout development (Townsend et al., 2012). Second, these results suggest that examining maternal effects on vocal production rates may give insight into how the early maternal environment impacts later social interactions. However, further studies are required to confirm these possibilities. Communication traits have so far received little attention compared to other metrics of social motivation across development. We advocate further studies in a range of species to investigate ontogenetic variation of vocal communication and the impact on social integration.

### Limitations of the study

In this study, we focused on cross-sectional data on wild chimpanzees in the Taï National Park, Ivory Coast. Even though our dataset is limited (52 immatures (0–10 years) and 36 mothers), we believe that, as this study required a huge logistical effort, this sample maximizes data per individual and per age group and thus the developmental insight we can gather from recordings of such detailed behavioral data in a natural setting.

## STAR★Methods

### Key resources table

**Table:** 

REAGENT or RESOURCE	SOURCE	IDENTIFIER
**Deposited data**		

Raw and analysed data	This paper	Figshare Data: https://doi.org/10.6084/m9.figshare.20481303.v1

**Experimental models: Organisms/strains**		

Individual subject details	See Table S1	N/A

**Software and algorithms**		

R studio version 3.6.1	R Core Team (2019)	https://cran.r-project.org/bin/windows/base/old/3.6.1/

### Resource availability

#### Lead contact

Further information and requests for resources should be directed to and will be fulfilled by the lead contact, Aisha C. Bründl (aisha.bruendl@gmail.com).

#### Materials availability

This study did not generate new unique reagents or genetic sequences.

### Experimental model and subject details

We studied three communities (East, North and South) of fully habituated western chimpanzees (*Pan troglodytes verus*) living in the Taï National Park (5°45′N, 7°07′W), Côte d’Ivoire. The North, South, and East communities have been studied since 1979, 1993, and 2000, respectively (Boesch et al., 2019; Boesch and Boesch-Achermann, 2000).

Our immature data set included 52 individuals (25 females and 27 males) aged between 4 days and 9.98 years collected across 8 years, from 2013 to 2020 (North community: 2017–2020, South community: 2013–2020, and East community: 2014–2020; see Table S1). In total, we collected 4326h of focal observations on immature chimpanzees (mean ± SE of 83 ± 12.6 h per individual). For this study, maternal data was collected during the following time period in the North community: 2009–2020, South community: 2004–2020, and East community: 2007–2020. The total number of focal observation hours across all 36 mothers was 16179h (mean ± SE 449 ± 29.7h per individual; see Table S1).

#### Ethics statement

Permissions to conduct the research were granted by the Ministère de l’Enseignement Supérieur et de la Recherche Scientifique and the Ministère de Eaux et Fôrests in Côte d’Ivoire and the Office Ivoirien des Parcs et Réserves. The Taï Chimpanzee Project is committed to non-invasive research and the protection of wild chimpanzees. Researchers followed a strict hygiene protocol (Wittig and Leendertz, 2015), including quarantine procedures for five days before observing the chimpanzees. In addition, researchers wear face masks and keep a minimum distance of eight meters to the chimpanzees to avoid disease transmission amongst other measures. Methods were approved by the “Ethikrat der Max-Planck-Gesellschaft”.

### Method details

#### Data collection

We collected detailed focal behavioural data (Altmann, 1974) of immature individuals and their mothers. We followed chimpanzees from dawn to dusk (from ca. 6.00 am to ca. 6.30 pm). We performed both full-day and half-day focal follows (Altmann, 1974), continuously recording the general activity and all social interactions of the focal individuals as well as the identity of the individuals in the party. An individual was determined as in the party if within visual range (usually 30–50 m) of the focal follower (and, implicitly, the focal chimpanzee) (Altmann, 1974; Mielke et al., 2017). Hereafter gregariousness refers to the number of individuals in the party of a focal individual. For half-day focal follows we switched to another focal individual at noon. We recorded every occurrence of pant-hoot vocalisations produced by the focal individual. A pant hoot was defined in our study as the focal individual having to produce a series of alternating pants and hoots with increasing volume. We considered both pant hoots with and without a climax phase as both call structures function as contact calls well beyond the range of visibility in a forest habitat (see: Girard-Buttoz et al., 2022a; Kalan et al., 2016). Each change in party composition was also documented. For all data collection, the team of local research assistants, PhD students and postdocs were trained by experienced trainers (Honora Kphazi, Grégoire N. Kohon, Liran Samuni and Roman M. Wittig). During each case of training, the trainees did not start collecting their own data until this reliably matched that of the trainer and additionally passed an inter-observer reliability test with the trainer from 2012 onwards (>80% agreement for two simultaneous, consecutive focal data recordings). Specifically, we checked if within a minute of one another the observers marked the same behaviour and that this behaviour included the same details (similarly to one-minute scan samples). All variables relevant for this study showed high inter-rater reliability (>80%) following an extensive training period, e.g., pant grunts, pant hoots and party composition (Boesch and Boesch-Achermann, 2000; Mielke et al., 2017, 2018; Wittig and Boesch, 2019a). We focused on individuals within this age range as after the age of 10 years, young chimpanzees become increasingly independent of their mothers and begin to integrate into dominance hierarchies (Pusey, 1983; Tkaczynski et al., 2020b), with females also emigrating soon after the start of adolescence (minimum observed emigration age in Taï = 10.66 years (Wittig and Boesch, 2019b)). As pant-hoot rates in adults can correlate with dominance (Mitani and Nishida, 1993), we avoided this confound in our analyses by focusing on immature individuals. We included eight orphans in our study for which we had information on the precise date when maternal loss took place (see Table S1). Our sample included orphans older than four years as chimpanzees are unlikely to survive without their mother before this age. We were concerned by the low observation time on certain individuals (minimum observation time per individual: 8.5h) so we conducted simulations to ensure that our sample did not comprise an unexpected number of observational zeroes and that the zeros were ‘true’ zeros (see Figure S1). These simulations also allowed us to select the best error distribution for our data and we used negative binomial Generalized Linear Mixed Models (GLMMs).

We also collated long-term data on each mother; namely gregariousness, age, pant-hoot rates, and dominance rank. As female chimpanzees were either adults when habituation started or they immigrate when they show genital swellings, we can’t be precise about their parity. Chimpanzees have not been shown to have reproductive senescence (Thompson et al., 2007) and there are no sterile females in our sample. We used genital swelling size (swellings are smaller during puberty) and immigration date as a proxy for age which relates to parity and maternal experience (Wittig and Boesch, 2019b). Also, the number of known offspring is not necessarily a good measure of parity due to relatively high infant mortality (Hill et al., 2001), hence age represents a potentially more informative measure of maternal experience. In Taï, the maternal experience of a female might not be the only factor that builds their maternal skills - observing other mothers might also be important (e.g., Carcea et al., 2021) and again age here would be a more relevant variable than parity. Dominance rank was estimated using a modified version of the Elo rating method ((Neumann et al., 2011) proposed by (Foerster et al., 2016)) using a maximum likelihood estimation of the gain factor (k parameter) and starting values for each individual (for details see: (Mielke et al., 2017)). We determined the Elo rating based on the directionality and number of pant grunts given amongst females, a unidirectional submissive signal given consistently from the lower to the higher-ranking individual (Wittig and Boesch, 2003). This maternal data was collected as for the immatures, based on focal follows conducted systematically since 1992, 1999, and 2007 in the respective North, South, and East communities (Wittig and Boesch, 2019a). 13 of the 36 mothers in our data set have multiple offspring (number of offspring per mother: mean 1.4 ±0.65SE, range: 1–3).

To investigate which ecological factors influence pant-hoot rates, we collected data on food availability and calculated a monthly food availability index based on three measures across the three chimpanzee territories: 1) the mean basal area (measured by trunk diameter at breast height) of tree species (phenology data), 2) the percentage of observed fruiting tree species with mature fruits each month within the territory of each group (for detailed methods see: Anderson et al., 2002; Valé et al., 2020), and 3) the density of tree species (habitat plot data; note, for this measure East territory data was unavailable and hence we used averaged data of the other two neighbouring territories) (Wessling et al., 2018).

For most individuals, the early-life socioecological data was extracted for each individual between birth and the end of the first two years of life. For individuals that were younger than two years at the time of sampling the endpoint of the early life window was the last observation date (see Table S1 for observation windows per individual). For instance, if an individual was sampled between the age of 1 and 1.5 years of age, the early life window of this individual was calculated during the 1.5 years after its birth. We chose the first two years of life to focus on early-life influences while maximising data per individual, as offspring are still fully dependent on their mothers. Although weaning typically occurs at 3–6 years of age (Lonsdorf et al., 2020), offspring older than two years have occasionally been known to survive after maternal death (Taï Chimpanzee Project, unpublished data) (Bründl et al., 2021). We considered the following maternal variables: (1) gregariousness - the average number of adults in the maternal party divided by the total number of adults in the community across the early-life window to make the data comparable across communities with different community sizes (taking into account the duration of a party); dominance rank - averaged across the early-life window (estimated using the directionality and number of pant grunts – see SOM); (2) age – number of days since the birth date of the focal immature; and (3) pant-hoot rate – the sum of pant hoots divided by the sum of observation time (in hours) across the early-life window. In addition, we defined orphan status as whether the focal was orphaned or not (after a minimum of 1 month after maternal loss to exclude any immediate effects of maternal loss and focus on the medium to long-term effects) at the time of sampling. Lastly, FAI was averaged across the early-life window (see Table 1). The current maternal factors were extracted on the day or across the year preceding each focal immature observation day depending on the detail of data collection available for each variable (see Table 1). This varying time period was to reduce measurement error by maximising data availability due to immatures and mothers being observed during different time periods.

### Quantification and statistical analysis

We conducted our statistical analyses in *R studio* version 3.6.1 (R Core Team, 2019). To investigate which socioecological factors (predictions 1–3) influence the number of pant hoots uttered per focal observation we ran two models: one model including all immature chimpanzees to test for early-life effects, incorporating orphans and non-orphans, and a second model only including non-orphans to see if current maternal effects were driving immature pant-hoot rates. The exact definition of each variable included in these two models can be found in Table 1.

In the first model (“early-life model”; N = 792 daily focal immature observations), we used the number of pant hoots produced by the focal on a given day as the response variable and included the following test predictors as fixed effects: focal age (in days) on the observation day, focal sex, and the following early-life factors (i.e., during the first two years of life): maternal gregariousness, age (as a proxy for maternal experience), pant-hoot rates, dominance rank and maternal loss (i.e., whether the immature’s mother immature died or was still alive, hereafter orphan status) as well as FAI (see above). Immature sex, early-life maternal predictors, and orphan status were included in an interaction with focal age to investigate whether the developmental trajectories of pant-hoot rates differed as a result of these potential socioecological modulators. The interaction term between age and early-life FAI was not incorporated in the models as the two predictors were highly correlated (0.63) and clustered by groups with older individuals and higher early-life FAI found in the East group compared to the North and South groups (see Figure S2). Besides the fixed predictor in our model, we accounted for repeated sampling on the same immatures, mothers with several offspring, group differences and variation in social and ecological parameters within group per year by incorporating focal identity, maternal identity, and a composite variable of year and group as random effects (e.g.: “2017_South”). Additionally, to tease apart the early versus current socioecological effects on immature pant-hoot rates (Mundry, 2014), we added current FAI, current number of siblings, and current number of adults in the party as control predictors in our model since they are all expected to influence calling rates (Cheney and Seyfarth, 2018; Fedurek et al., 2014; Stanton et al., 2017). To limit type I error rate at the nominal level of 5% (Schielzeth and Forstmeier, 2009), we included a maximum random slope structure (focal age, current FAI and number of adults in the current party within the three random effects). We also added the number of observation hours (log-transformed) during each focal day as an offset term to account for observation effort (McCullagh and Nelder, 1989). The log-transformation of the offset term was necessary to model the rate of pant hoots (i.e., number of pant hoots [response in our model] relative to observation time) in the log-linked space of the negative binomial models.

The second model (“current maternal model”; N = 595 daily focal immature observations) was identical to the first model except that we added the extra following current maternal factors – dominance rank, gregariousness, pant-hoot rate, and presence - which we did not have for the orphans in the first model to check whether any potentially significant early-life maternal predictors held after controlling for current maternal factors. The rationale behind this was that a previous study in this population found behavioural consistency within individuals and long-term repeatability for their social tendencies, such as gregariousness (Tkaczynski et al., 2020a). In this model, we used the current maternal factors as predictors and all other variables as control variables. We included the current maternal predictors as random slopes within each random effect in addition to those variables present already in the first model.

For both the “early-life model” and “current maternal model”, we used Generalized Linear Mixed Models (GLMMs (Baayen, 2008)) with negative binomial error structure and logit link function (see SOM for further detail on model choice) using the function “glmer” of the R package lme4 (version 1.121; (Bates et al., 2015) with the optimizer set to “bobyqa”). We confirmed that this error distribution was the best fit to our data using simulations (see SOM). To test the overall effect of the test predictors we compared the full models, comprising all the test and control predictors and the relevant interactions between them, with a null model comprising only the control predictors and the same random effects structure as the full model using a likelihood ratio test (LRT (Dobson and Barnett, 2018; Forstmeier and Schielzeth, 2011)). If the full-null model comparison was significant, we removed sequentially the non-significant interactions from the full model until obtaining a final model containing only significant interaction terms. The significance of each predictor (significance threshold set at p ≤ 0.05) in the model was assessed using the likelihood ratio test (LRT) run via the function “drop1” with argument ’test’ set to “Chisq”. We checked for variance inflation factors (VIFs) using the function “VIF” from the R package ‘car’ (Fox and Weisberg, 2019), which revealed that collinearity was not an issue (all VIFs <2.5 for the “early-life model” and <4.5 for the “current maternal model”) (James et al., 2014). We verified the assumptions of normally distributed and homogeneous residuals by visual inspection of QQ-plots (Field, 2005) and residuals plotted against fitted values (Quinn and Keough, 2002), which revealed no apparent issues. We assessed model stability by comparing the estimates obtained from the models based on all data with those obtained from models with each level of each random effect excluded one at a time (Nieuwenhuis et al., 2012). This showed that both models were relatively stable with no strong influence of any level of each random effect (i.e., no influential individual, mother or group-year). We calculated confidence intervals from parametric bootstraps using the “bootMer” function of the “lme4” package. In a final step, we calculated the effect sizes of the model as the portion of variance explained by the fixed effect (“marginal R^2^_m_”) and by the fixed and random effects combined (“conditional R^2^_c_”) (Nakagawa and Schielzeth, 2013) using the “r.squared GLMM” function from the “MuMIn’ package (Bartoń, 2020). To obtain comparable estimates for the predictors, and since we incorporated some interaction terms between some of these predictors, we standardized all continuous variables to a mean of 0 and a standard deviation of 1 in all models (Schielzeth, 2010).

## Data Availability

• All data are publicly available at Figshare. The DOI is listed in the key resources table. • This paper does not report original code. Custom R codes were used to analyse the data. All R codes and any additional information required to reanalyse the data reported in this paper are available from the lead contact upon request.
