# Supplementary material for: Maternal effects on the development of vocal communication in wild chimpanzees
Source: iScience. 2022 Sep 19;25(10):105152. doi: 10.1016/j.isci.2022.105152 (PMC9550609; doi:10.1016/j.isci.2022.105152)

iScience, Volume *25*

Supplemental information

Maternal effects on the development of vocal communication in wild chimpanzees

Aisha C. Bründl, Cédric Girard-Buttoz, Tatiana Bortolato, Liran Samuni, Mathilde Grampp, Therese Löhrich, Patrick Tkaczynski, Roman M. Wittig, and Catherine Crockford

Figure S1. Distribution of a simulated normal distribution of zeros a negative binomial GLMM can account for, related to STAR Methods. The red line depicts the number of zeros in our dataset.

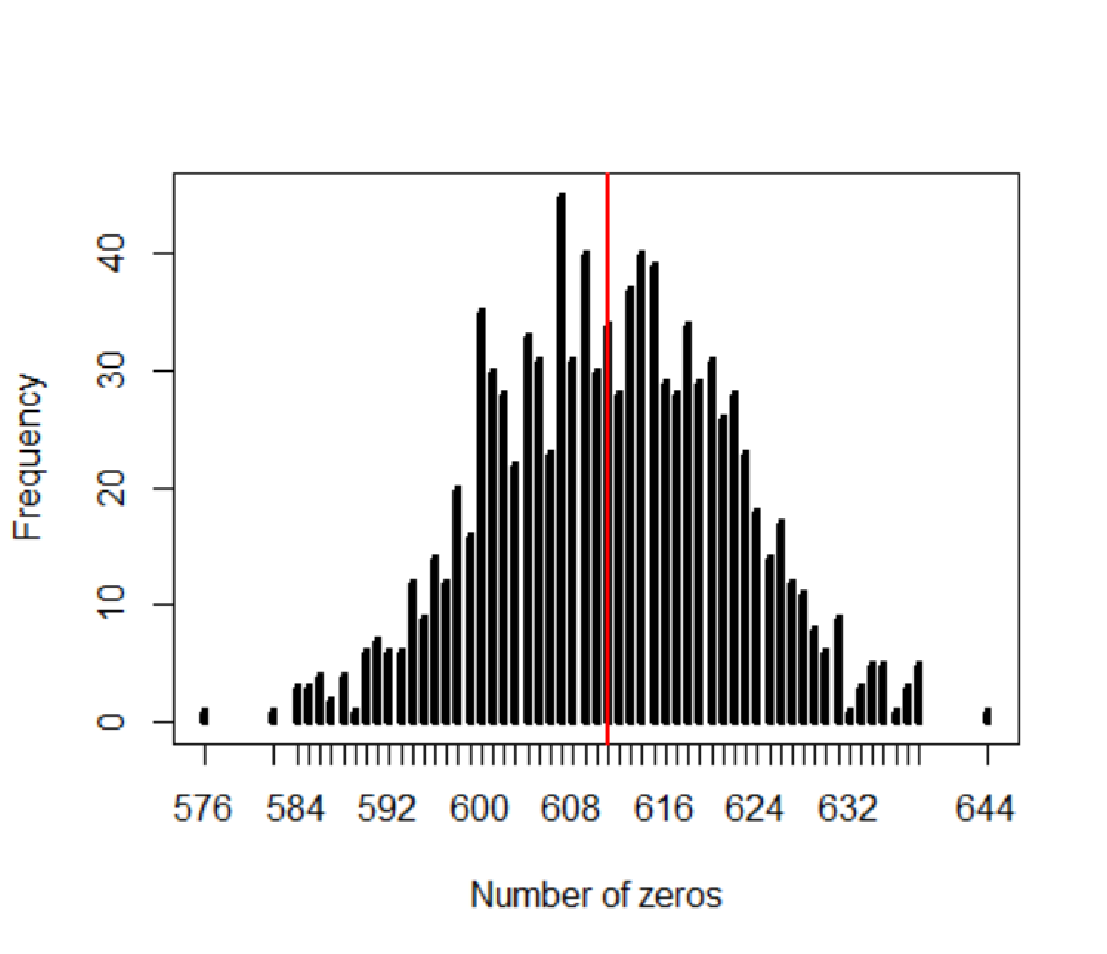

Figure S2. Distribution of (A) age (years) of the focal chimpanzees in this study and (B) early-life FAI across the three groups (East, North and South), related to STAR Methods. The boxplots are generated from raw data showing the median, interquartile range, minimum and maximum range, and outliers (N = 792).

(A) (B)

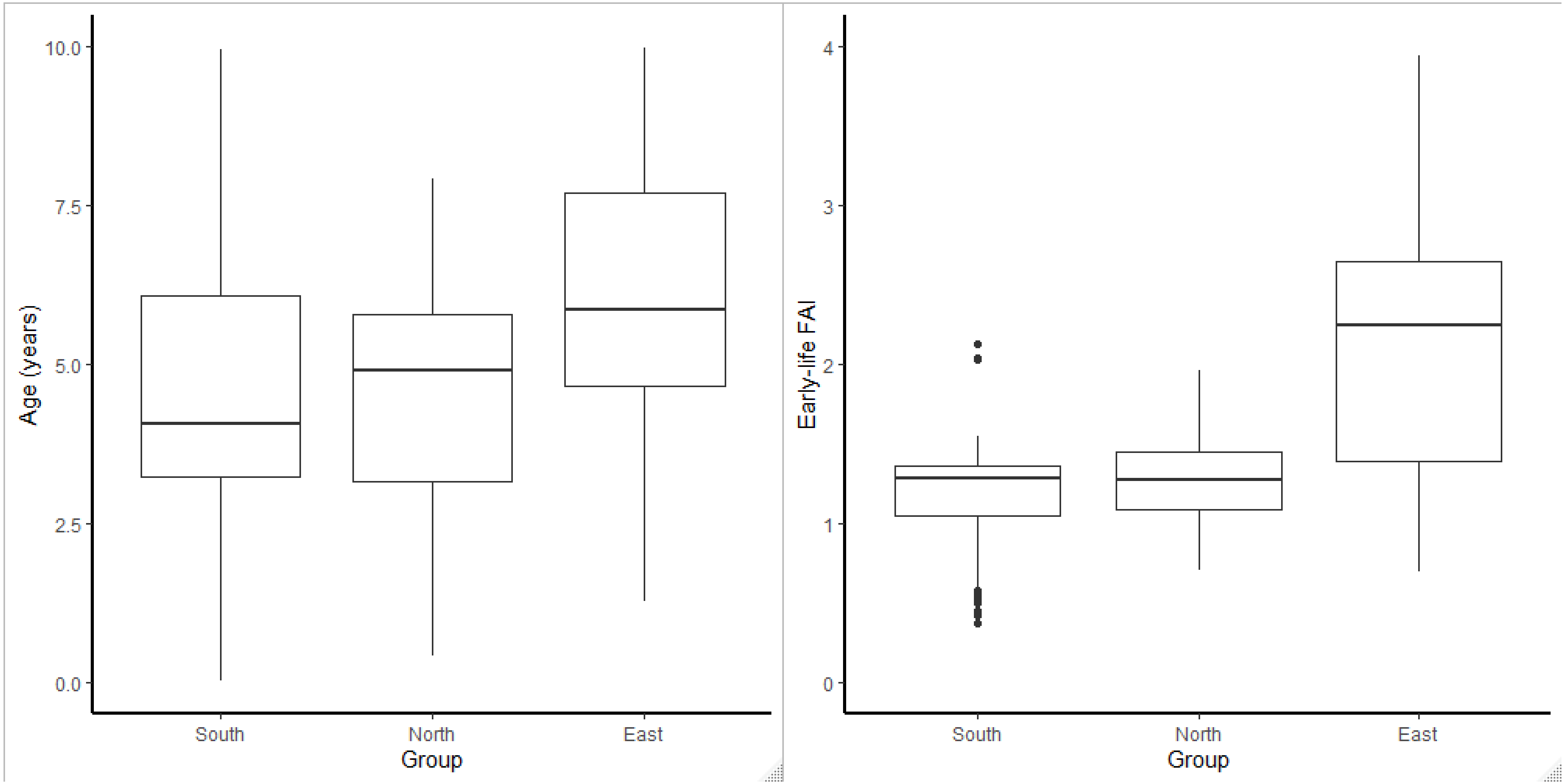

Table S1. Details of the individual subjects used in this study with associated sample sizes, related to STAR Methods. Sex (M/F), the identity of the Mother, Age (exact or estimated†) in years when orphaned (blank if not) and across which observations occurred (Minimum – Maximum), total Duration in hours of observational data, total Maternal duration in hours of observational data (Early and Current observation window) are reported for each subject. † We knew the exact birth date for most individuals (N = 40); for the other 12 individuals (see Table S1) we knew the month of birth. Some of the first observations were before the 15th of the birth month, thus we used the 1st day of the birth month as an estimated birth date in all cases where we did not know the exact birth date and calculated their ages accordingly.

Subject	Sex	Mother	Age (years)			Duration (hr)	Maternal duration (hr)	
			Orphaned	Minimum	Maximum		Early	Current
ChimpAEast	F	MotherAEast	4.9	4.6	8.8	293.8	277.0	43.2
ChimpBEast	F	MotherBEast	4.1	4.2	6.1	153.0	216.6	19.2
ChimpCEast†	M	MotherCEast	3.9	4.0	10.0	227.1	45.6	160.2
ChimpDEast	M	MotherDEast		3.8	6.5	52.5	306.6	31.2
ChimpEEast	M	MotherDEast		1.4	1.8	25.8	265.8	26.9
ChimpFEast†	M	MotherEEast		5.0	7.8	58.1	341.2	40.0
ChimpGEast	F	MotherFEast	5.4	5.5	5.6	23.8	53.4	8.1
ChimpHEast	M	MotherGEast		1.3	2.4	35.9	307.8	28.2
ChimpIEast	M	MotherGEast		6.2	10.0	232.2	19.7	69.5
ChimpJEast	F	MotherGEast		3.8	6.7	70.2	331.1	41.9
ChimpKEast	M	MotherHEast		1.8	1.9	21.7	328.3	21.6
ChimpLEast†	F	MotherIEast		2.5	3.7	30.5	395.8	28.7
ChimpMEast	M	MotherIEast		4.0	9.7	328.6	86.9	100.7
ChimpNEast	M	MotherJEast		1.3	2.4	23.4	179.9	25.1
ChimpOEast†	M	MotherJEast		7.3	10.0	69.3	32.0	40.8
ChimpANorth	F	MotherANorth		1.5	1.5	8.5	120.5	6.7
ChimpBNorth	F	MotherBNorth		4.8	7.8	64.1	380.4	212.8
ChimpCNorth†	M	MotherDNorth		3.3	3.4	17.3	554.3	4.8
ChimpDNorth	M	MotherCNorth		2.3	5.3	65.5	601.7	178.6
ChimpENorth	F	MotherDNorth		4.2	7.2	68.3	540.7	214.8
ChimpFNorth	F	MotherENorth		4.3	4.4	10.3	636.4	7.7
ChimpGNorth	F	MotherFNorth		3.3	6.3	61.5	622.8	194.9
ChimpHNorth†	M	MotherENorth		6.3	7.3	54.1	146.0	275.0
ChimpINorth†	F	MotherENorth		0.4	0.5	19.2	7.2	7.0
ChimpJNorth	F	MotherGNorth		4.9	7.9	59.2	132.5	172.6
ChimpKNorth	F	MotherGNorth		4.4	4.5	13.3	608.4	4.1
ChimpLNorth	F	MotherGNorth		0.4	0.5	37.1	5.8	4.1
ChimpMNorth	M	MotherHNorth		0.5	0.6	41.0	7.9	7.9

ChimpASouth	F	MotherASouth		3.3	6.3	67.2	65.4	68.0
ChimpBSouth	F	MotherASouth		2.0	2.8	27.2	300.5	13.9
ChimpCSouth	F	MotherBSouth	3.8	5.1	8.3	190.2	88.1	0.0
ChimpDSouth	F	MotherCSouth		1.7	2.6	28.6	276.0	24.4
ChimpESouth†	M	MotherDSouth		2.3	2.5	20.5	70.1	16.1
ChimpFSouth	M	MotherESouth		3.2	7.0	207.3	466.4	97.1
ChimpGSouth	M	MotherESouth		6.5	6.6	31.6	297.6	151.0
ChimpHSouth	M	MotherFSouth		2.3	4.5	62.9	288.5	66.2
ChimpISouth†	F	MotherGSouth		3.0	4.0	159.8	483.2	101.2
ChimpJSouth	F	MotherGSouth		3.3	4.1	24.4	440.5	20.1
ChimpKSouth	F	MotherHSouth		1.8	2.6	19.9	235.7	29.3
ChimpLSouth†	F	MotherHSouth		3.3	5.5	66.7	25.9	71.5
ChimpMSouth	F	MotherISouth	4.1	3.3	7.4	257.9	275.9	61.0
ChimpNSouth	M	MotherJSouth		2.1	3.0	23.9	234.1	19.5
ChimpOSouth†	M	MotherKSouth	4.8	8.7	9.9	179.3	281.3	0.0
ChimpPSouth	M	MotherLSouth		0.2	1.1	25.7	83.1	22.2
ChimpQSouth	M	MotherLSouth		2.8	5.1	71.1	363.3	87.4
ChimpRSouth	M	MotherMSouth		1.1	2.2	54.3	191.1	19.8
ChimpSSouth†	M	MotherNSouth		3.3	8.5	423.4	536.1	98.9
ChimpTSouth	M	MotherOSouth		0.2	1.2	25.9	67.3	15.2
ChimpUSouth	M	MotherOSouth		2.8	5.1	79.0	124.9	58.9
ChimpVSouth	F	MotherPSouth	4.5	8.8	9.3	64.9	75.2	0.0
ChimpWSouth	F	MotherQSouth		0.0	1.0	26.6	40.2	36.4
ChimpXSouth	M	MotherRSouth		1.5	2.4	22.8	234.1	28.4

Table S2. Current maternal effects on pant-hoot rates from a GLMM with negative binomial error structure and logit link function (N = 595), related to STAR Methods. Statistically significant effects ($P \leq 0.05$) appear in bold and the coded level of factors in parenthesis. *P* values are derived from likelihood ratio tests based on chi-square ($\chi 2$) values. X^2 and *P* values are not indicated in the first row because of having a very limited interpretation. All continuous predictors are z-transformed to a mean of 0 and a standard deviation of 1. Degrees of freedom are 1 for all predictors.

Predictors	Reference category	Estimate	± SE	Lower 95% CI	Upper 95% CI	*Z* value	X^2	*P* value
Intercept		**-2.842**	**0.296**	**-3.590**	**-2.327**	**-9.612**	-	-
Test predictors								
Focal age		**1.072**	**0.189**	**0.727**	**1.443**	**5.666**	**28.972**	**<0.001**
Sex (Male)	Female	**-0.706**	**0.312**	**-1.366**	**-0.124**	**-2.259**	**5.679**	**0.017**
Current maternal presence		0.035	0.105	-0.102	0.707	0.335	0.142	0.706
Current maternal gregariousness		-0.090	0.143	-0.409	0.182	-0.630	0.399	0.528
Current maternal dominance rank		0.116	0.216	-0.316	0.595	0.538	0.288	0.592
Current maternal pant-hoot rate		0.126	0.102	-0.169	0.339	1.243	1.381	0.240
Control predictors								
Current FAI		0.032	0.171	-0.311	0.312	0.187	0.036	0.851
Current party size		**0.287**	**0.134**	**0.012**	**0.507**	**2.136**	**4.837**	**0.028**
Current number of older siblings		0.105	0.168	-0.230	0.435	0.624	0.405	0.525

Figure S3. Maternal presence as a daily proportion the mother was present in the immature chimpanzee's current party with age (years), related to STAR Methods. The average daily proportion the mother was present was 0.98. The points show raw values (N = 595).

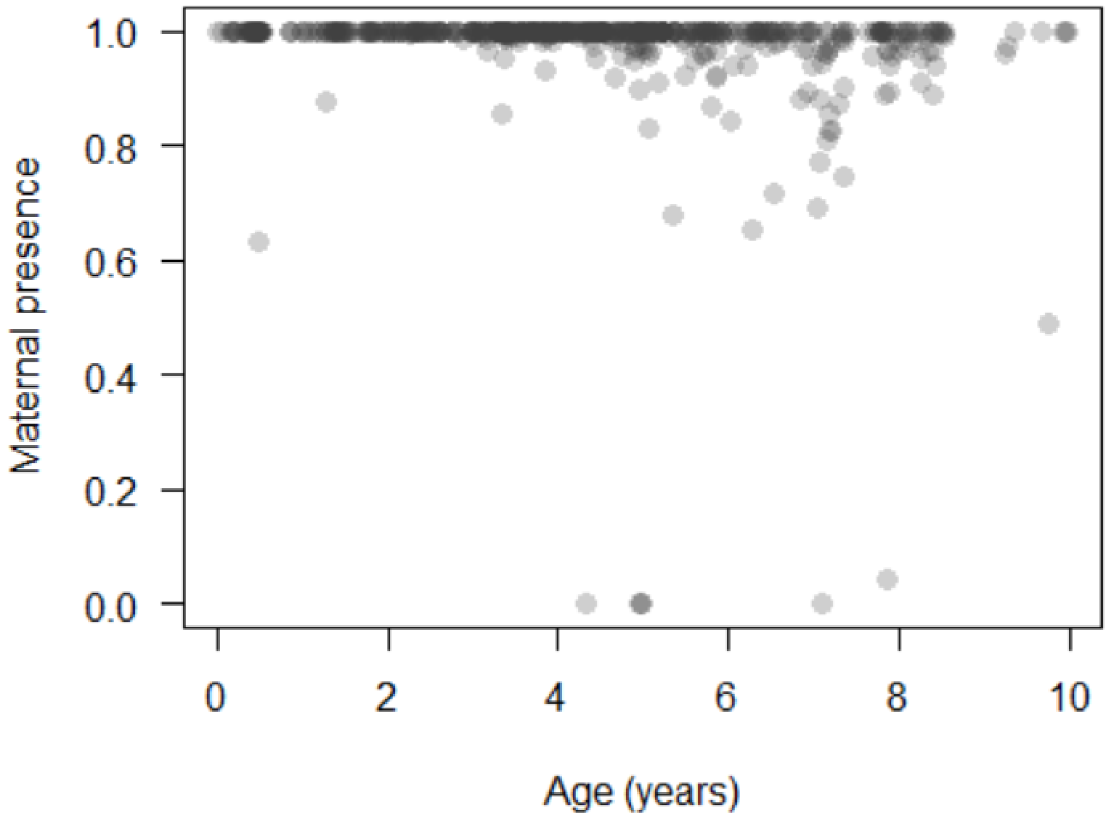

Supplement: Document S1. Figures S1–S3 and Tables S1 and S2 [file mmc1.pdf]
